# Multiple organ involvement in severe fever with thrombocytopenia syndrome: an immunohistochemical finding in a fatal case

**DOI:** 10.1186/s12985-018-1006-7

**Published:** 2018-05-30

**Authors:** Shibo Li, Yang Li, Qiujing Wang, Xuewen Yu, Miaomiao Liu, Haibo Xie, Liyong Qian, Ling Ye, Zhejuan Yang, Jianjing Zhang, Huimin Zhu, Wenhong Zhang

**Affiliations:** 1 Department of Infectious Diseases, Zhoushan Hospital, Wenzhou Medical University, Zhejiang, China; 2 Department of Infectious Diseases, Huashan Hospital, Fudan University, Shanghai, China; 3 Department of Infectious Diseases, Zhoushan Hospital, Zhejiang, China; 4 School of Public Health, Jining medical University, Jining, Shandong China; 5 Department of Critical Care Medicine, Maternal and Child Health Hospital, Zhoushan, Zhejiang, China; 6 Department of Pathology, Zhoushan Hospital, Zhejiang, China; 7 Daishan Centers for Disease Control and Prevention, Zhoushan, Zhejiang, China; 8 The First people’s hospital of Daishan, Zhoushan, Zhejiang, China

**Keywords:** SFTSV, Thrombocytopenia, Immunohistochemistry, Bunyavirus, Tick-borne, Emerging infectious diseases

## Abstract

**Background:**

Severe fever with thrombocytopenia syndrome (SFTS) is an emerging infectious disease caused by SFTS bunyavirus (SFTSV), a tick borne bunyavirus. However, Immunohistochemistry of SFTS patients are not well studied.

**Methods:**

We obtained multiple of tissues from a fatal case with SFTS, including blood, lungs, kidneys, heart, and spleen. The blood samples were used to isolate the causative agent for detection of viral RNA and further expression of recombinant viral protein as primary antibody. Immunohistochemistry of the heart, lungs, spleen and kidneys was used to characterize the viral antigen in tissue sections.

**Results:**

A 79-year-old man, together with his wife, was admitted because of fever. Both patients were diagnosed with SFTS by the positive SFTSV RNA in the blood. The gentleman died of multiple organ failure 8 days after hospitalization. However, his wife recovered and was discharged. Immunohistochemistry indicated that SFTSV antigens were present in all studied organs including the heart, kidney, lung and spleen, of which the spleen presented with the highest amount of SFTSV antigens. The kidney was next while the heart and lungs showed lower amount of SFTSV antigens.

**Conclusions:**

SFTSV can direct infect multiple organs, resulting in multiple organ failure and ultimately in an unfavorable outcome.

**Electronic supplementary material:**

The online version of this article (10.1186/s12985-018-1006-7) contains supplementary material, which is available to authorized users.

## Background

Severe fever with thrombocytopenia syndrome (SFTS) is an emerging infectious disease that was first reported in China in 2011 and then in South Korea and Japan [1–3]. SFTS bunyavirus (SFTSV), the causative agent of SFTS [3, 4], is mainly transmitted through tick bites, but occasionally from person to person through blood [5, 6], and rarely through aerosol [7]. SFTSV infects humans of all the ages, but predominantly those above 50 years old with mortality mainly occurring to those above 60 years, suggesting that the severity of SFTS is correlated to compromised immunity [8]. The mortality rate ranged from 7.9 to 50% in previous studies [1, 3, 8]. The incidence of the disease followed a trend of increasing annually [9].

The genome of SFTSV consists of three single-stranded negative sense RNA segments: S, M and L [3]. The M and L segments encode viral envelope glycoproteins and viral RNA polymerase, respectively. The S segment is an ambisense RNA that encodes a nonstructural protein (NSs) and a nucleoprotein (NP). The NSs protein of SFTSV has been reported to play pivotal roles in SFTSV replication and host responses [10].

The clinical manifestations of SFTS range from an acute febrile illness to multiple organ failure, encompassing fever, thrombocytopenia, leukopenia, gastrointestinal symptoms, and liver injury [3]. However, SFTSV viral protein expression in human tissues has been rarely studied. In light of these uncertainties, in this study we analyzed the viral NP expression in various tissues from autopsy in a lethal SFTV case by immunohistochemistry assays and indirect immunofluorescence assays with closely monitoring SFTSV viral load.

## Methods

### Ethics statement

The ethical committee of the Zhoushan Hospital has approved all human study and the study was conducted according to the medical research regulations of China. Written informed consent was obtained from the patients and their family members for this study. The animal experimental research in this study was approved by the bioethics committee of School of Public Health, Shandong University. All experiments were performed in accordance with relevant guidelines and regulations of China.

### SFTSV RNA load determination using quantitative real time PCR (qPCR)

Patients’ blood sample in heparin anticoagulant was collected on day 7 of hospitalization and RNA was extracted with RNeasy Purification Kits (QIAGEN, Germany). SFTSV RNA in the patient’ blood was detected by a qPCR. PCR primers and a probe were designed from a conserved region of the S segment of SFTSV, including forward primer P3: AGT TCA CAG CAG CAT GGA GAG GAT, reverse primer P4: ACT CTC TGT GGC AAG ATG CCT TCA, and a probe: FAM- TTG CTG GCT CCG CGC ATC TTC ACA TT –TAMRA. The qPCR was performed for one cycle at 95 °C for 15 s, 45 cycles at 95 °C for 5 s and 60 °C for 31 s.

### Genetic analysis

After extracting the viral RNA from the fatal case, the virus was sequenced with the use of the sequence-independent single-primer amplification (SISPA) method. Phylogenetic analyses were performed with the maximum likelihood method with the use of MEGA7 software.

### Viral detection using the immunohistochemistry assays and indirect immunofluorescence assays

#### Expression and purification of recombinant NP and obtaining primary antibody

The NP gene of SFTSV was amplified by reverse transcription PCR (RT-PCR) with the Access RT-PCR System Kit (Promega, Madison, WI). The sequences of the forward primer and reverse primer were: GAG GTA CCA TGT CAG AGT GGT CC and AAT CTC GAG TTA CAG GTT CCT GTA AG, respectively. The amplification conditions and parameters were as follows: one cycles at 45 °C for 45 min, 94 °C for 2 min, 40 cycles at 94 °C for 30s, 60 °C for 1 min, 68 °C for 2 min, one cycles at 68 °C for 7 min. The PCR product was cloned into pMD19 (Simple) T-Vectors (Clontech Laboratories, CA). The cloned insert was excised from the recombinant vector by double enzyme digestion and sub-cloned into pet-32a vector to express the NP. The recombinant NP was purified with the pET Express & Purify Kit—His60 (Clontech Laboratories, CA).

The purified recombinant NP was mixed with equal volume complete or incomplete Freund’s adjuvant (Sigma-Aldrich, USA) and injected subcutaneously into six 6- to 8-weekold Kunming mice (The Animal Experiment Center of Shandong University, Jinan City, China) to make polyclonal antibody. Each mouse was injected with 100 μg recombinant protein at multiple sites at one-week interval for 4 times. Mice were sacrificed 15 days after the last immunization to obtain sera. Sera were frozen at − 80 °C until use as the primary antibody.

#### Sample preparation

Autopsy tissues were obtained by puncture from the heart, lungs, spleen, liver, and kidneys of the fatal case in postmortem examine during the immediate 30 min following the death. Tissue slides were initially stained with Hematoxylin & Eosin (H&E) for morphological observations. Furthermore, tissue slides of heart, lungs, spleen, and kidneys were selected for immunohistochemistry with mouse antibody against recombinant NP of SFTSV. Paraffin embedded tissue sections were deparaffinized as described elsewhere [11]. Briefly, the sections were placed in a 60 °C incubator for 30 min. The sections were dewaxed with xylene and gradient ethanol and washed with phosphate buffer (PBS) and distilled water.

The sections were heat repaired in a container containing citrate buffer by heating with a microwave to keep the liquid temperature at about 98 °C for 10 to 15 min. The sections in the container were cooled down at room temperature for 30 min and then washed with PBS and dried by blotting. Hydrogen peroxide (3%) was added to the sections which were then incubated in a 37 °C water bath for 15 min to block the activity of endogenous peroxidase. After washing with PBS, the sections were dried by blotting.

#### Sampling labeling

One drop (30–50 μl) of the diluted mouse antibody to SFTSV recombinant NP as previously mentioned was added. Negative controls were added with normal mouse sera without the primary antibody correspondently. The sections were incubated at 37 °C for 1 h. The slides were rinsed with PBS. For the immunohistochemistry assays, the sections were incubated with rabbit anti-mouse horseradish peroxidase-labeled secondary antibody (1: 1000) (ZSGB-Bio, Beijing, China), and incubated at 37 °C for 1 h. The sections were stained with DAB coloring liquid (ZSGB-Bio, Beijing, China) and observed under a light microscope by two pathologists. For the indirect immunofluorescence assays, the sections were incubated with rabbit anti-mouse peroxidase-labeled secondary antibody (Alexa Fluor 488-conjugated Affinipure Goat Anti-Rabbit IgG, Proteintech, USA) (1:100 diluted), and then placed in an incubator at 37 °C for 1 h. After immunoreaction, nucleus staining with DAPI (C0065, Solarbio) was performed for 10 min at room temperature. The slides were washed again and the slides were examined under a fluorescence microscope (Olympus BX3).

## Results

### Case presentations

A couple was admitted into a local hospital on Zhoushan Island, Zhejiang Province, China because of fever with nausea and vomiting. Both two patients had no significant past medical history. Case one was the husband, 79-year-old male presenting with fever (38.5 °C), fatigue, diarrhea for 6 days, complicated with one episode of bright red hematemesis. On admission blood tests showed neutropenia, thrombocytopenia, with normal hemoglobin (Table 1). The patient was given hemostatic drugs, ceftizoxime and ribavirin after admission. Case two was the wife, 66-year-old. On admission, she reported fever (38.2 °C), fatigue, nausea, and anorexia. Blood test showed slightly low white blood cells (WBC), thrombocytopenia, and slightly anemia (Table 1). She was given lansoprazole, levofloxacin, rehydration and symptomatic treatment. Further questioning revealed that both patients had tick bites on the face and legs about 1 week before onset of illness. Therefore, SFTS was highly suspected for this couple. The decision was made to determine the SFTSV viral load and samples from the couple tested positive for SFTFV.

**Table 1:** Laboratory test results of patients

	Day after admission									
		1	2	3	4	5	6	7	8	9
Days after onset	P1^a^	6	7	8	9	10	11	12	13	14
	P2^b^	2	3	4	5	6	7	8	9	10
PLT (× 10^9^/L)	P1	89	58	35	20	11	10	14	62	69
	P2	83	^c^ND	44	39	28	28	38	44	67
WBC (×10^9^/L)	P1	1.3	1.1	0.7	3	4.4	3.9	6.5	11	15.4
	P2	4.1	ND	2	2	1.5	1.9	2.1	2.2	2.3
HB (g/L)	P1	138	135	134	126	114	81	77	50	41
	P2	130	ND	124	127	119	116	120	117	111
AST (U/L)	P1	93	131	650	650	862	852	703	719	922
	P2	ND	ND	45	48	49	39	38	31	40
ALT (U/L)	P1	38	40	171	164	223	168	102	83	166
	P2	ND	ND	17	24	27	25	24	23	26
ALB (g/L)	P1	33.8	32.4	27.6	25.9	26.3	26.3	23.5	21.7	17.4
	P2	ND	ND	37.1	37.7	35.8	37.4	39.3	43.9	40
LDH (U/L)	P1	240	337	965	1134	1418	1649	2511	–	4840
	P2	ND	ND	218	295	286	282	267	241	246
CK (U/L)	P1	499	665	1151	1261	1451	1126	2337	–	3783
	P2	ND	ND	217	255	207	149	97	62	52
CKMBU/L)	P1	17	19	42	50	51	74	92	–	125
	P2	ND	ND	8	11	8	11	8	6	5
CR (umol/L)	P1	83.2	101.7	87	85	84	107	153.6	152	232
	P2	ND	ND	75.2	65.7	57.7	52.9	68.2	54.1	55.7
BUN (mmol/L)	P1	6.9	7.33	5.2	7.8	6.41	7.8	10.33	9.6	12.6
	P2	ND	ND	4.43	3.23	2.68	2.12	2.51	3.17	3.89
APTT (s)	P1	ND	41.6	49.3	54.7	90.5	97.9	116.2	78.1	76.4
	P2	ND	ND	34.1	31.2	31.7	30.5	29.5	28.9	27.2
PT (s)	P1	ND	12.3	13.3	11.8	11.4	10.4	10.8	11.7	14.2
	P2	ND	ND	12.1	11.2	11.1	10.8	11.2	11.2	11.4
D-D (ng/ml)	P1	ND	983	3110	4522	2581	1210	392	389	409
	P2	ND	ND	608	572	412	401	418	415	390
Potassium (mmol/l)	P1	3.88	3.71	3.28	3.4	3.36	3.48	3.62	3.9	4.13
	P2	ND	ND	3.02	3.6	3.37	3.45	3.52	3.74	4.3
Sodium (mmol/l)	P1	127	129.9	132.6	129.9	134.0	130.1	138.4	139.0	138
	P2	ND	ND	132.6	140.6	139.4	142.4	142.1	137.4	143

^a^*P1* patient 1, ^b^*P2* patient 2, ^c^*ND* not done

**Fig. 1:**
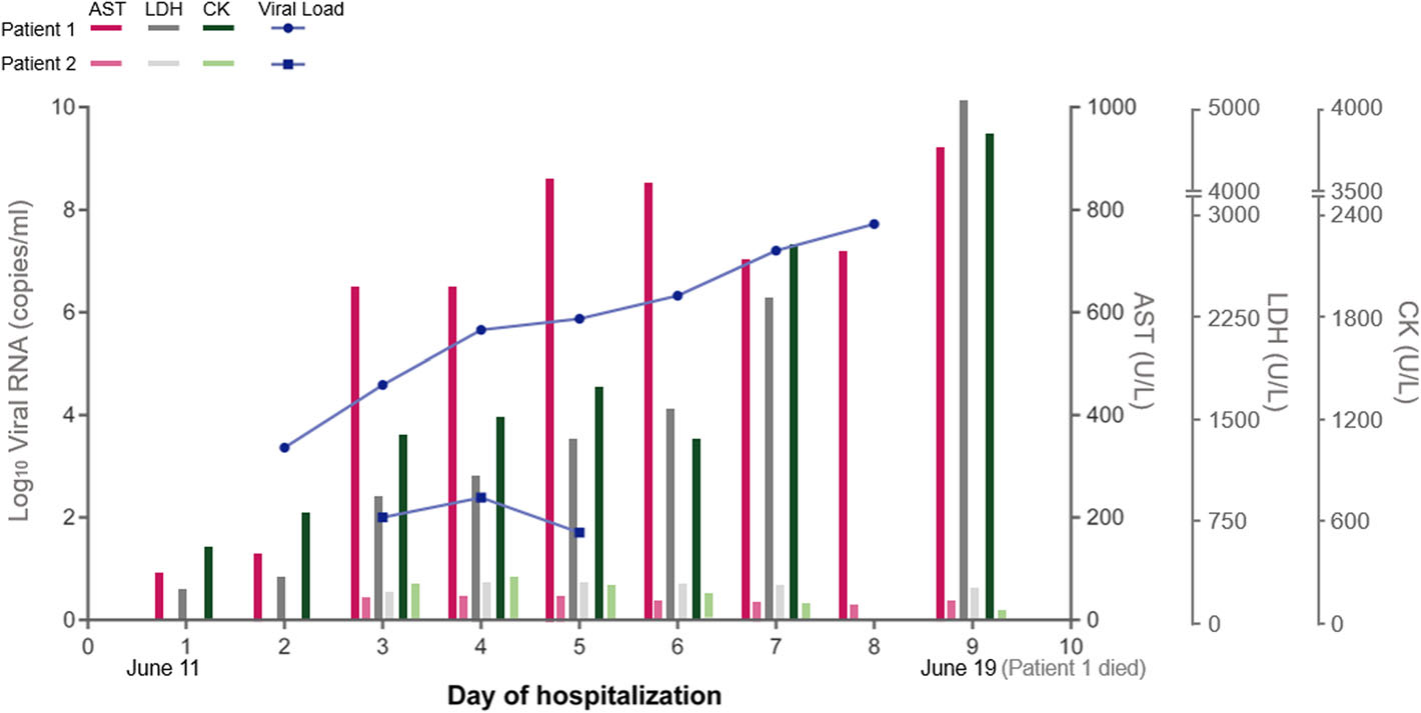
The clinical course of two SFTS patients. The condition of Patient 1 deteriorated rapidly and he died at day 9 of hospitalization while Patient 2 was in a relatively stable condition. Patient 1 revealed a higher SFTSV viral load, higher levels of AST, LDH and CK than his wife (Patient 2). AST: Aspartate transaminase; LDH: lactate dehydrogenase; CK: creatine kinase

After admission, Case one experienced continuous progressive increase of aminotransferase, lactate dehydrogenase (LDH), creatine phosphokinase (CK), and serum SFTSV viral load (Fig. 1) and decrease of platelet and serum albumin, prolonged activated partial thromboplastin clotting time (APTT) (Table 1). On day 5 after hospitalization, the patient became delirium and unconsciousness and had oral mucosal bleeding, crackles in the lungs, right lower extremity ecchymosis, and respiratory failure. An APACHE II (Acute Physiology and Chronic Health Evaluation II) score was seven points and a SOFA (sequential organ failure assessment) score was four points. The patient was given tracheal intubated on mechanic ventilation, plasma exchange, blood filtration, red blood cell transfusion, anti-viral and antibacterial drugs, albumin, and fibrinogen. His symptoms still did not resolve. On day 7 after hospitalization, the patient developed coma with sluggish pupillary light reflex and unstable vital signs. Two days later, the patient died.

Case two was conscious but listless without bleeding, skin rash, jaundice, or lymphadenopathy. The patient had scattered rales in the lungs. Laboratory test results showed that aminotransferase, LDH, CK and viral load were mildly increased (Fig. 1) and thrombocytopenia and leukocytopenia were further observed; serum potassium and sodium ions were decreased slightly (Table 1). Bone marrow biopsy showed hemophagocytic phenomenon. The patient was treated positively and her condition gradually returned to normal. The patient recovered and was discharged on the sixteenth day after admission.

### SFTSV viral load of the patients

SFTSV viral load was closely followed up for 7 days for both patients. On the second day after hospitalization, case one was serum positive for SFTSV RNA by qPCR amplification. On day 3 after hospitalization case two also turned into serum positive for SFTSV by qPCR. Case one had much higher viral load and longer period of SFTSV viremia than case two and the viral load of case one had been continuously increasing with the extension of the disease until death (Fig. 1).

### Laboratory results of the patients

Laboratory examination showed that PLT, WBC, and hemoglobin decreased in both patients. The level of aspartate transaminase (AST), LDH, and CK was dramatically elevated in the fatal patient (case one). Unremarkable change (LDH) or no change (AST, CK) was observed in mild patient (case two) (Fig. 1). The level of D -dimer was significantly high in the fatal case during the entire course of illness, but only slightly increased in the mild patient for 2 days. Prolonged APTT was only presented in the fatal case. These results suggested multiple organ failure and presence of DIC in the fatal case.

### Microscopic morphological findings

**Fig. 2:**
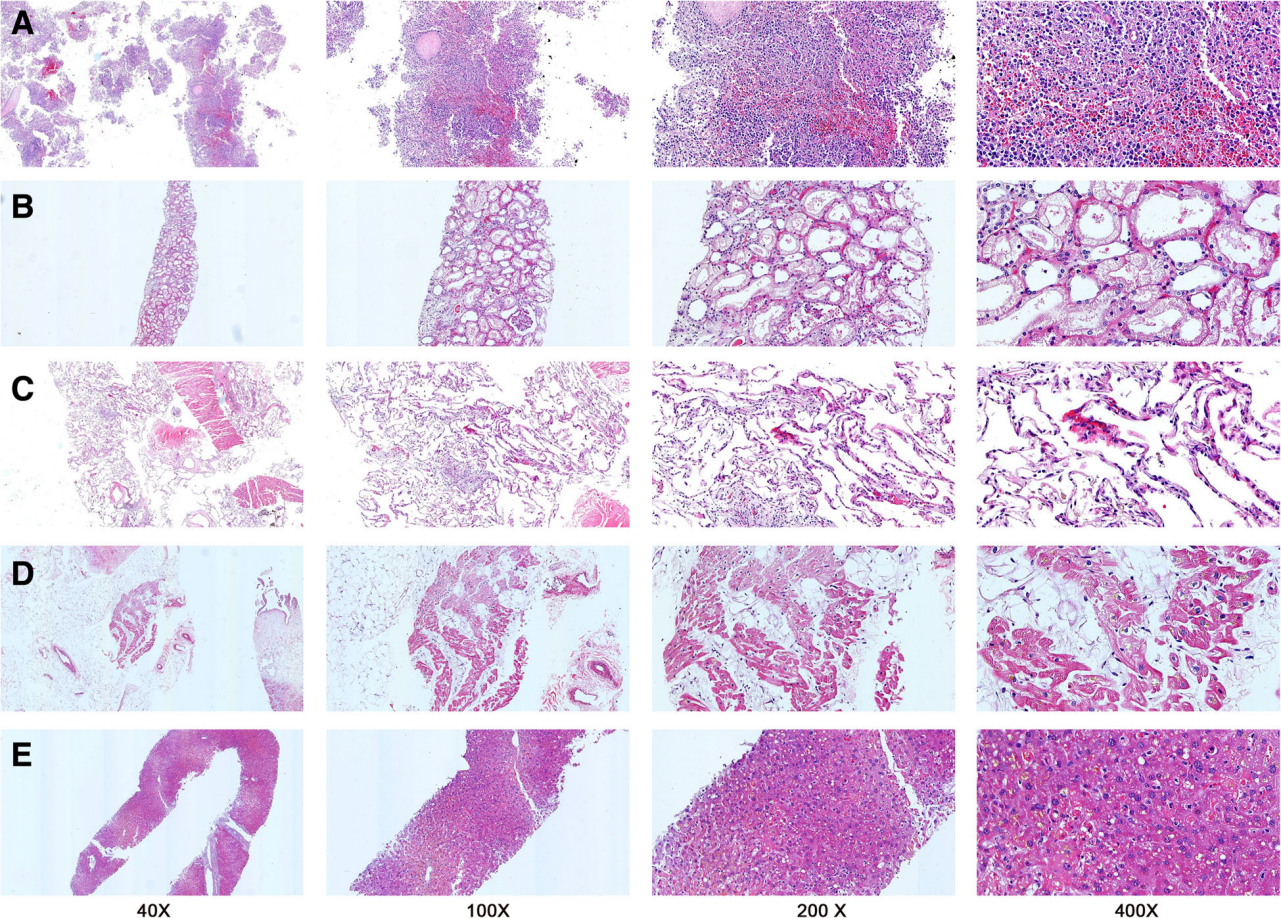
The microscopic morphological observation using Hematoxylin & Eosin stained slides of a decreased SFTSV patient. SFTSV infections involved multiple organs including the spleen (Panel **a**), kidneys (Panel **b**), lungs (Panel **c**), heart (Panel **d**) and liver (Panel **e**)

Findings of H&E staining sections showed congestion and focal hemorrhage in the spleen. Ischemic lesions were also observed (Fig. 2a). The kidney was microscopically eroded with dilated tubules where swollen renal tubular epithelial cells were seen (Fig. 2b). The alveolar spaces were flooded with edema fluid and interstitial fibrous proliferated (Fig. 2c). A small amount of expansion of capillary could be observed in several organs including kidneys and lungs. Myocardium cells revealed structural disorders with vacuolar degeneration, with lipofuscin dispersed (Fig. 2d). Liver histological changes could be found as well, such as expansion of portal area, congestion in hepatic sinusoid, and acidophilic degeneration (Fig. 2e).

### Immunohistochemistry assays and indirect immunofluorescence assay results

**Fig. 3:**
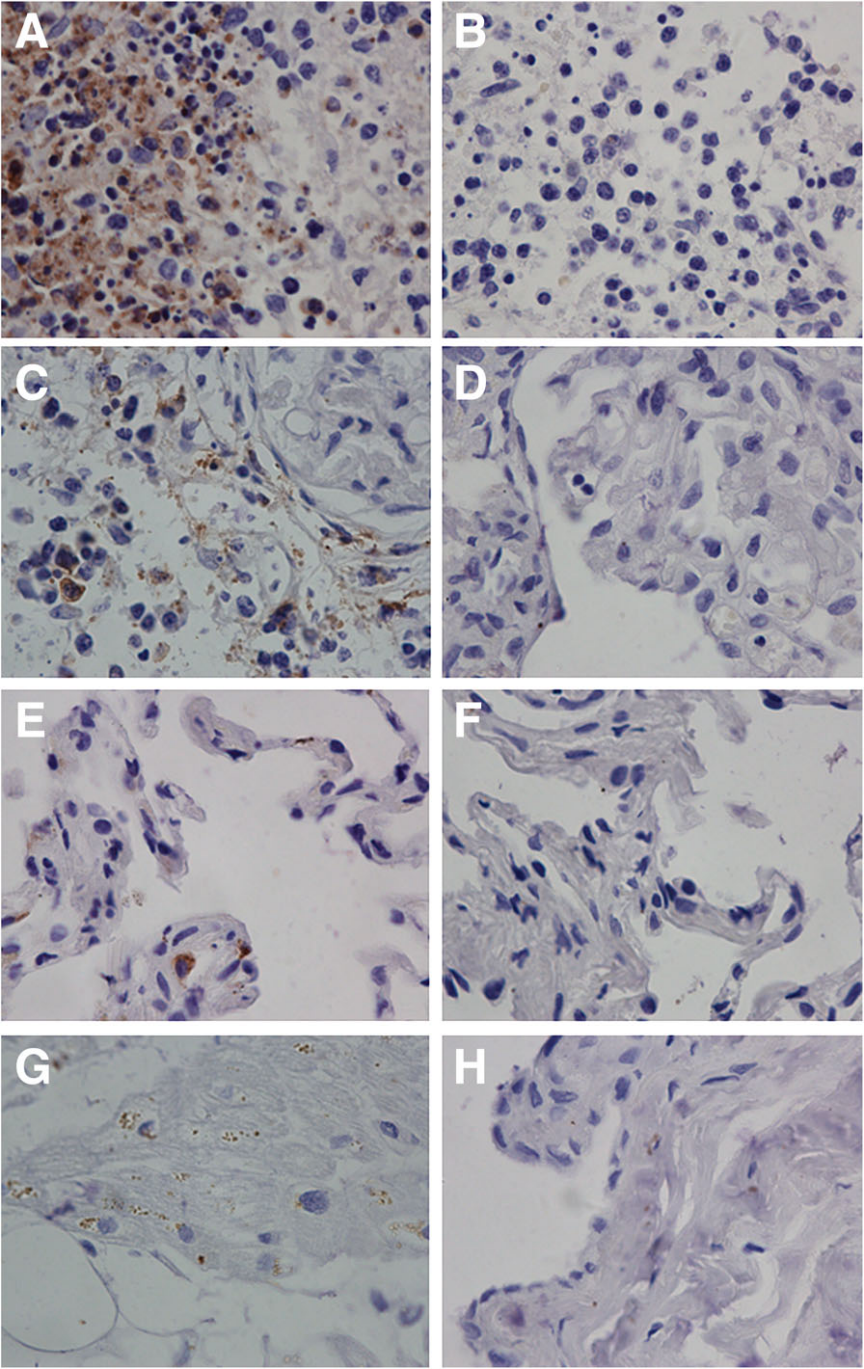
Immunohistochemistry results of a deceased SFTSV patient. The spleen tissues had the most amounts of SFTSV antigens (Panel **a**); the kidney had moderate amount of SFTSV antigens (Panel **c**), and the lung (Panel **e**) and the heart (Panel **g**) had least amount SFTSV antigens. Negative controls were stained by omitting the primary antibody incubation using the spleen (Panel **b**), kidneys (Panel **d**), lungs (Panel **f**), heart (Panel **h**) tissue sections respectively. (original magnification × 400)

**Fig. 4:**
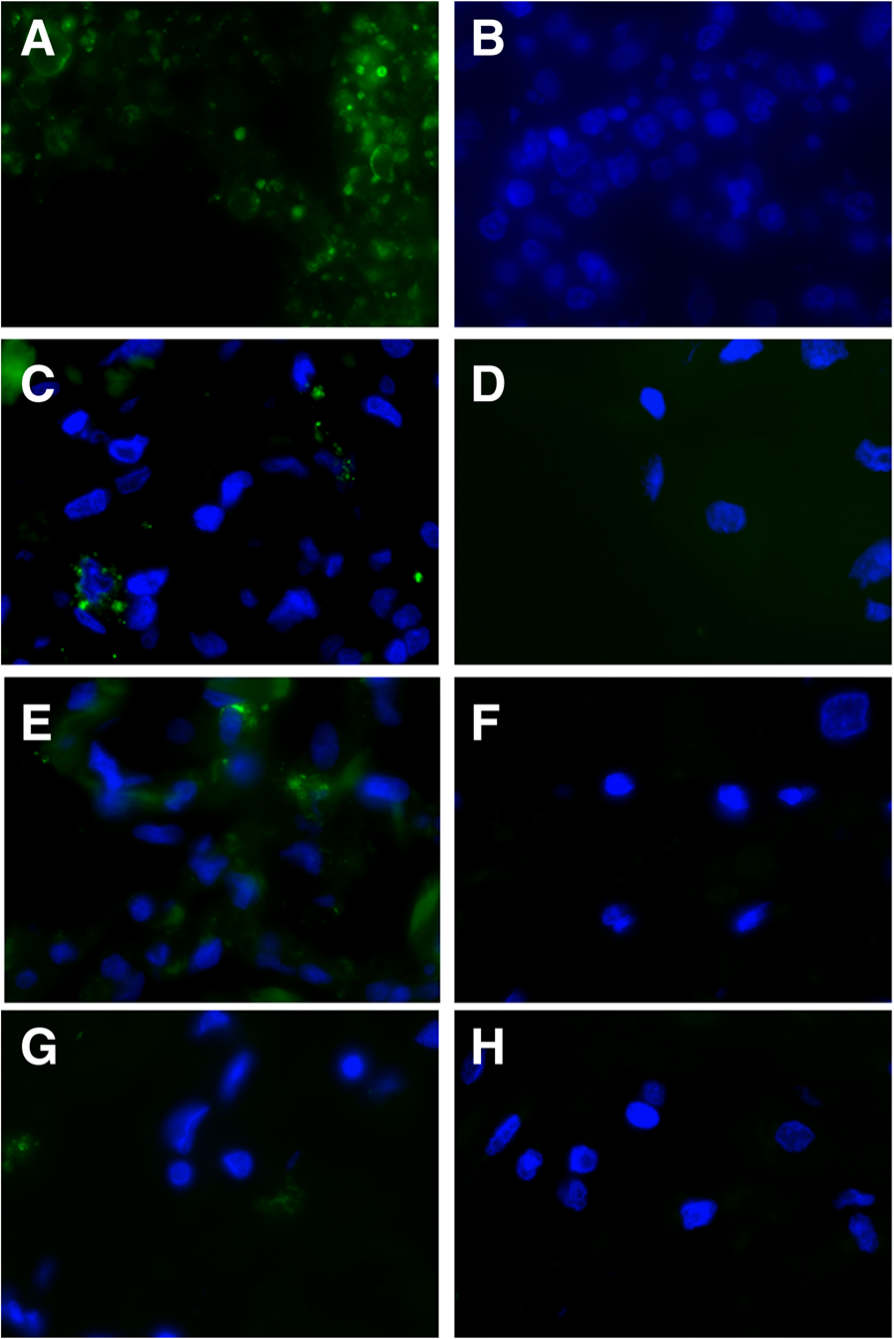
Immunofluorescence assays results of a deceased SFTSV patient. The high immunofluorescence staining of SFTSV antigens within the spleen was shown (Panel **a**) while the median immunofluorescence staining of SFTSV antigens within the kidneys (Panel **c**), lungs (Panel **e**), and the low immunofluorescence staining of SFTSV antigens within the heart (Panel **g**). Corresponding negative controls showed no immunofluorescence staining (Panel **b**, spleen; Panel **d**, kidney; Panel **f**, lungs, Panel **h**, heart) (original magnification × 400)

Immunohistochemistry studies showed a positive staining for SFTSV NP in sections from all organs tested including the spleen, kidneys, lungs, and heart (Fig. 3a, c, e, g) while negative staining corresponded to the controls specimen (Fig. 3b, d, f, h). Furthermore, the SFTSV antigens predominantly exhibits a cytoplasmic pattern. The SFTSV antigens were the most widespread and abundant in the spleen, especially in the white pulp. The sections from the kidneys also revealed the viral antigens expressing in the glomeruli. Compared to the spleen and the kidneys, the virus antigens in the heart and lungs was detectable despite much lower abundance. Meanwhile, the immunofluorescence assays showed the spleen, kidneys, lungs, and heart tissue were positive in SFTSV antigens (Fig. 4). The discrepancies in antigens between different organs were comparable with that appeared in the immunohistochemistry assays.

### Molecular characterization

**Fig. 5:**
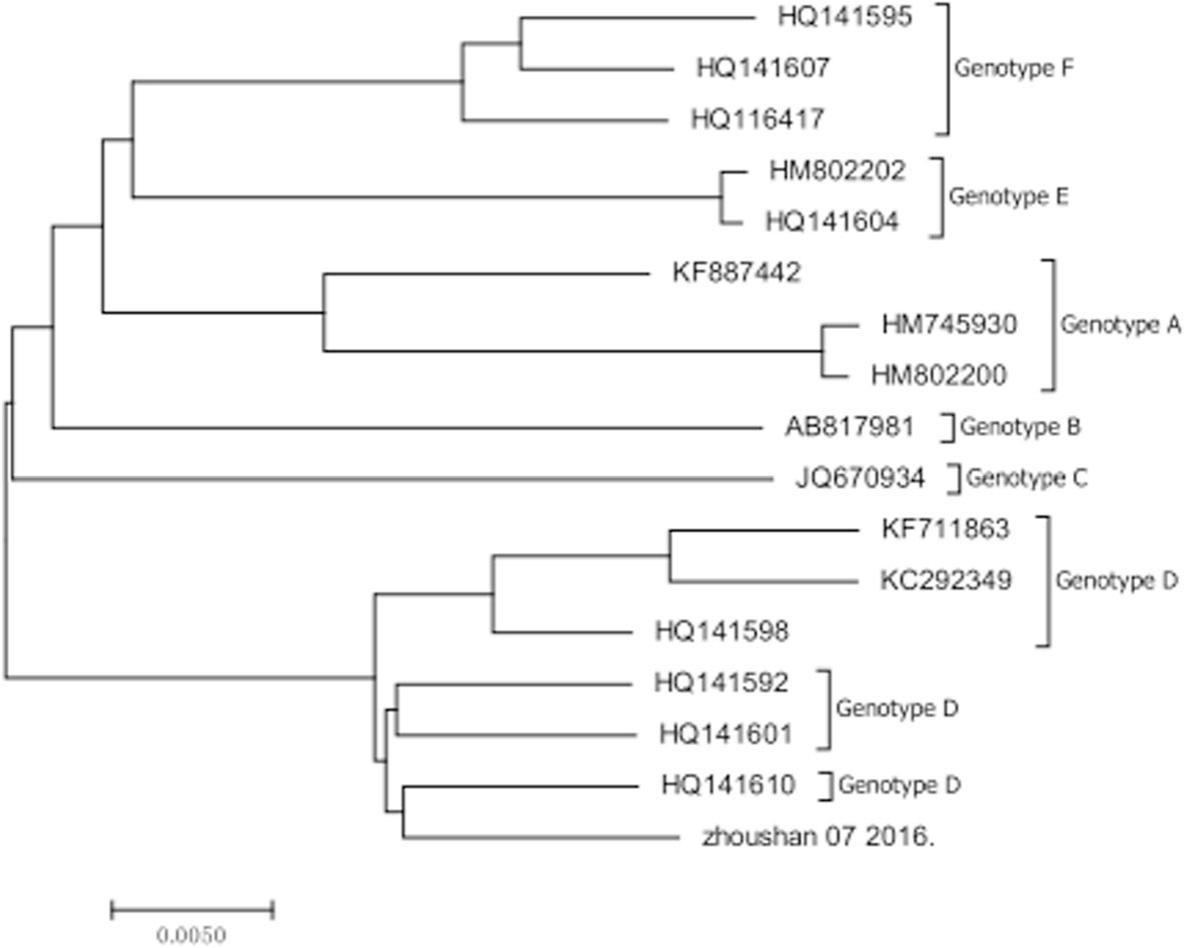
Phylogenetic analysis of the isolate from the fatal patient and other SFTSV. The phylogenetic tree was generated from complete nucleotide sequence of L segment

The whole genome of the fatal patient’s SFTSV isolates was completely sequenced. We are able to yield the full sequence of three viral segments, including L segment of 6368 nucleotides, M segment of 3378 nucleotides and S segment of 1744 nucleotides respectively (see Additional file 1). Phylogenetic trees based on complete viral genome sequence of L segment showed the SFTSV isolate clustered well with other known SFTSV isolates (Fig. 5). And the sequence date also suggested that case one was infected by genotype D SFTSV.

## Discussion

We reported the clinical manifestations and laboratory tests of a fatal SFTS patient and a mild SFTS patient in a cluster. Viral immunohistochemistry assays and indirect immunofluorescence assays of multiple tissues were examined in the deceased patient. Tick bites were rarely reported in SFTS patients usually with an unknown incubation time. These two patients had a clear date of their tick bites and onset of illness, consisting with the incubation time around 5 to 8 days since the contact.

The fatal case suffered from severe coagulopathy with diffused bleeding of GI tract and skin, ended up with delirium, and coma. The fatal patient had higher liver enzymes, LDH, and CK than the mild patient who recovered from the disease. The fatal patient also had coagulation dysfunction with prolonged activated partial thromboplastin time and elevated D-dimer levels than the mild patient. Previous study indicated that elevated AST, LDH, CK and CK-MB were risk factors associated with severity among SFTS patients and fatality among severe SFTS patients [12].

High serum viral load has been considered to be a high-risk factor that resulted in the death of SFTS patients [13]. Our study further confirmed that the serum viral load was correlated with the severity of the disease. Therefore, monitoring viral load might assist in evaluating the prognosis of the disease. Although the pathogenesis of SFTS remains elusive. The host immune system is essentially indispensable for the pathogenesis of SFTSV infection, in addition to the high level of virus replication. Some studies suggested that cytokine mediated inflammatory response is characterized by the imbalance of cytokines and chemokines, and plays an important role in the progression of SFTSV infection [12, 14]. Previous reports have showed SFTSV antigens in lymph nodes, liver, spleen, bone marrow and adrenals, but not in the heart, lungs, kidneys, gastrointestinal tract, aorta, or iliopsoas muscle [1, 15, 16]. Although we failed to perform the IHC assay for the liver tissue due to overmuch liver tissues loss when trimmed and liver tissue sections falling coming off slide, the initial immunohistochemistry and immunofluorescence findings still broadened the knowledge of the extent of SFTS that SFTSV infection is not only limited in the spleen, but also extensively involves the kidneys, the lungs, and the heart. Our study is consistent with a mouse model of SFTSV infection, which indicated that SFTSV primarily infects the spleen and lymph nodes [17]. These studies suggested that SFTSV could infect most organs of the patients, with heaviest infection in the spleen.

## Conclusions

SFTSV virus was found in multiple organs, with the highest viral load in the spleen, moderate load in kidneys, and the least in the lung and heart. In addition, SFTS patients with higher viral load and higher liver enzymes, LDH, and CK indicated severity of the disease and even fatal outcome; the incubation time of SFTS was about 5 to 8 days after tick bite.

## Additional file

Additional file 1: The DNA sequence information from the SFTSV isolated from the fatal diseases. (DOCX 17 kb)

## Abbreviations

APTT: Activated partial thromboplastin clotting time
AST: Aspartate transaminase
CK: Creatine phosphokinase
LDH: Lactate dehydrogenase
NP: Nucleoprotein
NSs: Nonstructural protein
PBS: Phosphate buffer
qPCR: Quantitative real time PCR
RT-PCR: Reverse transcription PCR
SFTS: Severe fever with thrombocytopenia syndrome
SFTSV: Severe fever with thrombocytopenia syndrome bunyavirus
SISPA: Sequence-independent single-primer amplification
WBC: White blood cells
