# Supplementary material for: Multiple organ involvement in severe fever with thrombocytopenia syndrome: an immunohistochemical finding in a fatal case
Source: Virol J. 2018 May 30;15:97. doi: 10.1186/s12985-018-1006-7 (PMC5977472; doi:10.1186/s12985-018-1006-7)
Supplement: Supplementary file 1 — The DNA sequence information from the SFTSV isolated from the fatal diseases. (DOCX 17 kb) [file 12985_2018_1006_MOESM1_ESM.docx]

**Complete L segment 6368bp**

ACACAGAGACGCCCAGATGAACTTGGAAGTGCTTTGTGGTAGGATAAACGTGGAGAATGGGCTGTCTCTTGGAGAACCAGGCCTGTACGACCAAATCTACGACAGGCCTGGGCTACCAGACCTAGATGTGACTGTCGATGCCACAGGTGTGACAGTAGACATAGGGGCTGTGCCAGACTCAGCATCACAATTGGGTTCATCAATCAATGCTGGGTTGATCACAATCCAGCTCTCTGAAGCATATAAGATCAATCATGACTTCACGTTCTCTGGCCTGTCAAAGACAACAGATCGACGCCTCTCAGAGGTGTTCCCCATTACCCATGATGGTTCTGATGGGATGACCCCTGATGTGATTCACACCAGATTAGATGGGACCATTGTGGTGGTTGAATTTTCAACCACTAGGAGCCATAACATTGGGGGCCTGGAGGCAGCATATAGGACAAAGATAGAAAAATATAGGGACCCAATCTCAAGGCGTGTTGATATTATGGAGAACCCGAGGGTCTTCTTTGGCGTTATTGTAGTCTCGTCAGGAGGGGTTCTGTCCAACATGCCCCTGACTCAGGATGAGGCAGAGGAGCTCATGTACAGGTTTTGCATTGCCAATGAGATCTACACTAAGGCTAGATCTATGGATGCAGACATTGAGCTACAGAAGAGTGAGGAAGAGCTTGAGGCTATTAGCAGGGCACTATCATTCTTCAGTTTGTTTGAGCCTAACATTGAAAGAGTAGAAGGAACATTCCCTAATTCAGAGATCGAGATGCTGGAACAGTTTCTCTCAACACCAGCTGATGTTGACTTCATCACCAAGACCCTCAAAGCAAAAGAGGTAGAGGCTTATGCTGATCTTTGTGACAGCCACTACCTAAAGCCTGAAAAAACCATTCAGGAGCGGCTAGAGATCAATAGATGTGAGGCTATTGACAAAACTCAGGACCTCCTAGCTGGTCTACATGCAAGGAGCAACAAGCAAACATCATTGAATCGAGGGACAGTCAAGCTCCCGCCCTGGCTACCAAAGCCGTCAAGTGAGTCAATAGACATCAAGACCGACTCAGGCTTTGGTTCCTTAATGGATCATGGCGCATATGGTGAGCTGTGGGCAAAGTGCCTTCTAGATGTCTCGCTGGGCAATGTGGAGGGGGTAGTCAGTGACCCTGCAAAAGAACTTGACATTGCTATCTCTGATGATCCAGAAAAAGATACCCCCAAAGAGGCAAAGATAACCTATAGGCGATTCAAGCCTGCCTTAAGTTCAAGTGCCCGTCAAGAATTTTCTCTCCAAGGAGTGGAGGGGAAGAAGTGGAAGAGAATGGCAGCAAACCAGAAGAAAGAGAAGGAGTCCCATGAAGCATTGAGCCCTTTCTTGGACGTTGAAGACATTGGGGATTTCCTAACATTCAACAATCTTCTTGCAGATTCAAGGTATGGGGATGAGTCCGTCCAGAGAGCAGTGTCAATCTTGTTGGAGAAGGCATCTGCCATGCAAGACACAGAGCTCACTCATGCCCTCAACGATTCATTCAAGAGGAACCTAAGCAGTAATGTGGTTCAGTGGTCCCTTTGGGTCTCCTGTTTGGCACAGGAGCTAGCTAGTGCTCTGAAGCAGCACTGCAGGGCTGGTGAGTTTATCATCAAGAAGCTGAAGTTCTGGCCTATCTATGTCATTATCAAGCCGACCAAATCATCATCCCATATCTTCTACAGCTTAGGGATCCGCAAGGCTGACGTGACAAGGAGGCTCACTGGCAGAGTCTTCTCTGATACCATTGATGCTGGGGAATGGGAGTTAACAGAGTTCAAAAGCCTGAAGACATGCAAGCTTACAAACCTTGTCAACTTGCCATGCACCATGCTGAACTCAATAGCTTTCTGGAGAGAGAAGCTGGGCGTGGCTCCATGGCTGGTTCGAAAACCTTGTTCAGAGCTCAGGGAGCAGGTGGGCCTGACCTTCCTGATCAGCCTGGAGGACAAGTCTAAGACTGAGGAGATCATCACCTTAACAAGGTACACCCAGATGGAGGGCTTTGTCTCTCCCCCCATGCTGCCTAAGCCCCAAAAGATGCTAGGGAAACTGGATGGACCTTTGAGAACTAAGCTACAGGTATACCTCCTCAGGAAGCATCTGGATTGCATGGTGCGAATTGCTTCTCAGCCATTCAGCCTAATCCCTAGAGAGGGGAGGGTAGAATGGGGAGGAACATTCCATGCCATCTCAGGCAGGTCCACAAACCTTGAGAATATGGTGAACAGCTGGTACATTGGGTACTACAAGAACAAAGAGGAGTCAACAGAGCTAAATGCCCTCGGAGAAATGTATAAGAAGATCGTGGAGATGGAAGAGGACAAGCCCAGCAGCTCTGAGTTTCTAGGGTGGGGGGACACTGATTCCCCTAAGAAGCATGAATTCTCACGGAGCTTCCTCAGAGCTGCTTGCTCATCTCTGGAGAGAGAAATTGCTCAGAGACATGGAAGACAATGGAAGCAGAACCTTGAGGAGCGTGTCCTGAGAGAGATTGGGACCAAGAACATTCTGGACCTTGCATCCATGAAGGCCACAAGCAACTTTTCCAAAGACTGGGAGCTCTACTCAGAAGTCCAGACCAAAGAGTACCATAGGTCCAAACTATTGGAGAAGATGGCCACATTGATTGAGAAAGGGGTCATGTGGTACATTGATGCTGTGGGCCAGGCATGGAAGGCAGTTCTAGATGATGGGTGCATGCGAATCTGTCTCTTCAAGAAGAATCAGCACGGTGGCCTCAGAGAGATCTACGTTATGGATGCGAATGCCCGGCTTGTGCAGTTTGGGGTTGAGACCATGGCTAGGTGTGTCTGTGAGCTGAGCCCACATGAGACTGTTGCCAACCCTAGGCTCAAGAATTCCATCATAGAGAACCATGGGCTGAAGTCAGCCCGTAGTCTTGGCCCAGGCTCTATAAACATAAACTCATCCAATGACGCCAAGAAGTGGAATCAGGGGCACTACACAACAAAGCTAGCTCTAGTTCTTTGTTGGTTCATGCCAGCCAAATTCCACAGATTCATTTGGGCTGCCATTTCCATGTTTCGGAGAAAAAAGATGATGGTGGACCTAAGGTTTTTAGCTCACCTCAGTTCTAAATCTGAGTCCAGGTCATCTGATCCGTTCAGGGAAGCAATGACAGACGCCTTCCATGGTAATAGGGAAGTCTCATGGATGGACAAAGGGCGAACTTACATAAAGACAGAGACAGGGATGATGCAGGGCATACTGCACTTTACATCCAGTCTCCTCCACTCTTGTGTTCAGAGCTTTTACAAGTCTTATTTCGTCTCGAAGCTCAAAGAGGGCTACATGGGGGAAAGCATCAGTGGGGTGGTGGACGTCATAGAAGGTTCTGACGACTCTGCGATCATGATCAGCATACGCCCTAAGTCAGACATGGATGAAGTCCGATCAAGGTTTTTTGTTGCTAACTTGCTCCACTCTGTCAAGTTCTTAAACCCTTTGTTTGGGATTTATTCATCAGAGAAATCAACAGTGAACACAGTGTATTGTGTCGAGTATAACTCTGAATTCCATTTCCACAGGCACTTGGTTAGACCCACACTGAGATGGATAGCAGCGTCTCACCAAATCTCAGAGACTGAAGCCCTTGCAAGCAGGCAAGAGGATTACTCTAACCTTCTGACCCAGTGCTTGGAAGGAGGGGCCTCATTCTCTCTAACCTACCTCATACAGTGCGCTCAGCTCCTACACCACTACATGCTTCTAGGACTATGCTTACATCCCTTGTTTGGAACCTTCATGGGGATGCTGATATCAGACCCAGATCCAGCCCTAGGGTTCTTCCTCATGGACAATCCTGCATTCGCAGGGGGAGCAGGATTCAGATTCAATCTGTGGAGAGCCTGCAAGACTACAGACCTTGGGCGAAAGTATGCATATTATTTCAATGAGATACAGGGTAAAACAAAGGGAGATGAGGACTACAGGGCTCTGGACGCCACATCGGGAGGAACTCTCAGCCACTCTGTTATGGTGTATTGGGGGGACAGGAAGAAGTATCAGGCCTTATTGAACAGGATGGGCCTTCCTGAAGACTGGGTGGAGCAGATAGATGAGAATCCTGGAGTCCTTTACAGGAGAGCTACCAACAAGAAGGAACTACTCTTAAAGCTGGCAGAGAAGGTTCATTCACCTGGTGTGACTAGCAGCCTGAGTAAAGGGCATGTGGTACCTCGGGTGGTGGCAGCAGGAGTATACCTCCTCTCTCGCCACTGCTTCCGCTTTAGTTCAAGCATCCATGGCAGGGGCTCAACGCAGAAGGCTAGCCTTATAAAACTGTTGATGATGTCTTCTATTTCTGCCATGAAGCACGGGGGCTCACTAAACCCTAATCAGGAGCGAATGCTCTTCCCTCAGGCTCAAGAGTATGACAGGGTATGCACATTGCTTGAGGAAGTTGAACACCTAACAGGGAAATTTGTTGTTAGGGAGAGGAACATTGTCAGGAGCCGCATAGACTTGTTCCAAGAGCCAGTGGACTTGCGGTGCAAGGCAGAAGATCTGGTGTCTGAGGTGTGGTTTGGCCTGAAAAGGACTAAGCTTGGGCCCCGTCTCCTCAAGGAAGAGTGGGACAAACTTAGGGCCTCATTTGCATGGTTGAGCACAGACCCATCTGAAACATTGAGGGATGGTCCTTTTCTTAGCCATGTGCAGTTTAGGAACTTCATAGCCCACGTTGATGCCAAATCAAGATCAGTCAGGCTCCTAGGTGCCCCCGTGAAGAAGTCAGGCGGGGTCACCACTGTAAGCCAAGTAGTCAGAATGAACTTCTTCCCTGGTTTTAGCCTAGAGGCTGAGAAGAGCTTAGACAATCAGGAAAGACTTGAGAGCATCTCCATCCTCAAGCATGTCTTGTTCATGGTCTTGAATGGCCCTTACACTGAGGAGTACAAGCTGGAAATGATCATAGAGGCCTTCTCTACTCTTGTGATACCACAGCCATCAGAGGTCATCAGGAAATCAAGGACCATGACTTTATGCCTTTTATCGAATTACTTGTCTAGTAGGGGTGGGTCCATTCTAGACCAGATTGAGAGGGCACAGTCAGGCACTCTAGGAGGATTCAGCAAGCCCCAGAAGACATTCATTAGGCCAGGAGGTGGTGTTGGCTACAAGGGAAAAGGTGTGTGGACTGGAGTGATGGAGGACACCCATGTCCAAATTTTGATAGATGGAGATGGGACTAGTAACTGGCTTGAGGAGATCAGGCTCAGCAGTGATGCCAGGCTTTATGATGTCATTGAATCCATCCGAAGGTTATGTGATGACCTTGGGATCAACAACAGGGTAGCATCTGCATATAGAGGTCATTGCATGGTTAGGCTGAGTGGATTCAAGATCAAGCCAGCATCAAGGACTGACGGCTGTCCAGTCAGGATTATGGAAAGGGGCTTCAGGATTAGGGAACTCCAAAACCCAGATGAAGTCAAGATGAGAGTGAGGGGCGACATCCTTAACCTCTCTGTCACTATACAAGAAGGAAGGGTCATGAACATTCTAAGCTACAGGCCGAGAGACACTGACATATCAGAGTCAGCCGCAGCATACCTCTGGAGCAATCGAGACCTCTTCTCCTTTGGGAAGAAGGAGCCATCCTGCAGCTGGATCTGCTTGAAGACTCTTGACAATTGGGCCTGGTCACATGCCTCAGTTCTCCTGGCAAATGATAGGAAGACCCAAGGCATTGACAATAGAGCTATGGGGAACATCTTCAGGGACTGTCTCGAGGGTTCTCTAAGAAAGCAAGGGCTGATGAGGTCAAAGCTCACAGAGATGGTTGAGAAGAATGTAGTTCCTTTAACAACCCAAGAGCTTGTCGACATACTGGAGGAGGACATTGACTTTTCAGATGTCATAGCTGTAGAGCTCTCAGAGGGATCACTTGACATTGAATCCATCTTTGATGGAGCACCTATCTTGTGGTCTGCTGAGGTGGAAGAGTTTGGAGAAGGAGTGGTGGCTGTGAGCTATTCCAGTAAGTACTATCATCTAACCCTGATGGACCAAGCTGCCATCACAATGTGTGCAATCATGGGTAAGGAAGGGTGTAGAGGGCTCCTTACTGAGAAGAGATGCATGGCAGCCATACGAGAGCAGGTACGGCCATTCCTCATATTCCTGCAAATTCCTGAGGACAGTATTTCTTGGGTATCTGATCAGTTCTGCGACTCCAGGGGTCTTGATGAAGAGAGCACCATTATGTGGGGTTAACTTTAAAACACGGTTGGTACGCAGCTGATGTGTCTGTGGGTGACTAGGGAATGTTGGTTTTAGAAGGATTTCCTTAAGATCTGGGCGTTCTTTGTGT

D

ACACAAGAACCCCCAAAAAAGGAAAGACGCAAAGGAGTGATCATGTCAGAGTGGTCCAGGATTGCAGTGGAGTTTGGTGAGCAGCAGCTCAATTTGACTGAGCTTGAGGATTTCGCGAGAGAGCTGGCCTATGAGGGCCTTGATCCTGCTTTGATCATCAAGAAGCTGAAGGAGACAGGTGGAGATGATTGGGTGAAGGATACGAAGTTCATCATTGTCTTTGCTCTGACTCGAGGCAATAAGATCGTCAAGGCATCAGGGAAAATGTCAAACTCAGGGTCTAAGAGGTTGATGGCACTCCAAGAGAAATATGGATTGGTTGAGAGGGCAGAAACCAGGCTCTCAATCACTCCTGTGAGGGTAGCGCAGAGCCTTCCCACCTGGACATGTGCAGCAGCAGCAGCCTTAAAGGAGTATCTCCCAGTGGGGCCAGCCGTCATGAACCTGAAGGTTGAGAATTATCCTCCTGAGATGATGTGCATGGCCTTTGGATCCCTGATTCCAACTGCGGGGGTATCTGAAGCTACAACCAAGACCCTGATGGAGGCCTACTCTCTGTGGCAAGATGCCTTCACCAAGACCATCAATGTGAAGATGCGTGGAGCCAGCAAGACAGAAGTTTACAACTCCTTCAGGGACCCTCTCCATGCTGCTGTGAACTCTGTCTTCTTTCCCAATGATGTTCGGGTGAAGTGGCTGAAGGCCAAGGGAATCCTTGGCCCAGATGGGGTTCCCAGCAGAGCTGCTGAGGTTGCTGCTGCTGCTTACAGAAACCTGTAAGCAGCAATTTGACATATCCAATTAGGCTAAATTCCATGATCCAATTCGACAAAATTAGACCTCCTTCGGGAGGTCACCAATGGCTGGCCAATCTTTTCCCCAAATGGTGTTCAGGGCTGTAGTTCTCAATTCTTCATCAAACCTGATGAGGTTGTGGAATGGGCACCTATTTAAATAGTGTGCCATCAGATATGGGTAACCCTGGAGGACCCCAGGCTCATCATCCTCATCCAAGACACTGTATGAGCCATCTGTCTTCTTTTTGCGGGTAGGAATGAAGAAATCCCAACAGTCTAACAGAGGCTTACGCAATGAGGAAGAAGTGAACAAGTGGTGGTTCAAGCTCATGCCTCTTAGTCTAGCAAGTGACTCTATCTGGAGGAGGGCCACATCCAGAATTGGGGATAGTCCAGTTATTAGGATCTCAGGCAATCCAAGTCTAAGTCTTCGCTTCTCTATGGCTTCAAGAGAATCCAATATCATATCCCCTTCAAAAAGATACTTTTGGCCTTTTGTTATCCTGTGGAAGAGGCCCCGCCAGTTCTCTCTTCCGGTTTCCATTGCCCAGCTGTTCTTGATGGAGAAGAACATGTGAGCTGCCTGTAGGAACCATACAGATGGCTTCCCACTAGGCCACCTAAGAGCACTCAGTTCTCTCTGGGAGTAGCACCTCATGTCCTTGTAGTACATGTCTAACAGCTGCTCAAGTCCAGGCTCAAGCTGATGAAGAGGATTTTTTGGGTTGCCAAACAACCATACTGTGTTGGTCATCTTGCCCATCCTCTTGACCATTGACAAAGTCAACACACTACAAGAGCATTCAACAAGATCTCTCCTAAGAGTGGGATACTCTCCCAGGGAGGGCTCAAGCCTAACAGTGTTAGCATTCACTGCTACAGATTTGAGGTCAACGTTGGAGCATTTGCTCAGCGACATGGTTTCCAAATGAAGGGGGTCTTTGTGT

Complete M segment

ACACAGAGACGGCCAACAATGATGAAAGTCATCTGGTTCTCCTCTCTGATCTGCTTAGTCATTCAATGCGGTGGGGATACGAGCCCAATCATCTGCGCAGGACCCATCCACTCAAACAAGAGTGCTGACATACCCCACCTGCTTGGCTACTCTGAGAAGATTTGTCAGATAGATCGGCTGATACATGTTTCGTCATGGCTTAGAAACCACTCACAATTTCAGGGCTACGTAGGGCAGCGAGGTGGACGCTCTCAGGTGAGCTACTACCCAGCTGAAAATTCCTACTCAAGGTGGAGTGGACTTCTAAGCCCCTGTGATGCTGATTGGCTTGGGATGCTTGTTGTGAAGAAGGCCAAGGGGTCTGATATGATAGTTCCTGGGCCTTCATACAAGGGGAAAGTCTTTTTTGAACGGCCAACTTTTGATGGATACGTAGGCTGGGGCTGTGGTAGTGGGAAGTCTAGGACTGAGTCAGGTGAGCTCTGCAGTTCAGACTCTGGGACTAGTTCTGGTCTTCTGCCCTCGAATAGGGTTCTCTGGATAGGTGATGTTGCTTGTCAGCCTATGACACCCATCCCTGAGGAGACATTTCTGGAGCTGAAGAGCTTTAGCCAAAGTGAATTCCCTGACATATGCAAAATTGATGGCATTGTGTTCAACCAGTGTGAGGGTGAGAGTCTACCTCAGCCCTTTGATGTTGCGTGGATGGATGTTGGCCACTCTCATAAAATCATCATGAGGGAGCACAAGACCAAATGGGTACAAGAGAGCTCATCCAAGGATTTTGTGTGCTACAAGGAAGGGACTGGGCCTTGTTCTGAATCAGAAGAAAAGACTTGCAAGACCAGTGGATCATGCAGGGGGGACATGCAGTTTTGCAAGGTGGCAGGTTGTGAACATGGGGAAGAGGCATCTGAAGCCAAGTGTAGATGCTCACTTGTGCACAAGCCCGGAGAAGTCGTTGTGTCATATGGAGGGATGCGTGTTAGACCAAAGTGCTATGGTTTCTCCAGAATGATGGCAACACTGGAGGTGAACCAACCAGAGCAAAGGATTGGTCAATGCACTGGCTGCCATCTAGAATGCATAAGTGGGGGTGTGAGGCTGATCACTCTAACCAGTGAGCTCAAGTCAGCTACTGTCTGCGCTTCTCACTTTTGTAGTTCTGCCACAAGTGGTAAGAAAAGCACGGAGATTCAATTCCACTCAGGATCATTGGTTGGGAAAACAGCAATCCACGTCAAAGGGGCACTGGTGGATGGAACTGAATTCACATTTGAGGGTAGTTGCATGTTTCCAGATGGTTGTGATGCAGTGGACTGCACATTCTGTCGTGAGTTTCTAAAAAATCCTCAGTGCTACCCTGCAAAGAAGTGGCTGTTCATCATTATTGTCATCCTCCTTGGATATGCAGGCCTCATGCTACTCACCAATGTTCTTAAGGCAATTGGGGTTTGGGGGTCATGGGTCATAGCTCCAGTGAAGCTAGTGTTTGCCATCATAAAGAAGCTAATGAGAGCTGTGAGCTGCCTGATGGGGAAATTGATGGATAGGGGAAGGCAAGTGATCCATGAGGAAATAGGGGAGAATAGAGAGGGCAACCAAGATGATGTTAGGATCGAGATGGCCAGACCCAGGAGGGTAAGGCATTGGATGTACTCGCCTGTCATCCTGACTATTCTAGCAATAGGGCTTGCTGAGGGCTGTGATGAAATGGTCCATGCTGATTCAAAACTTGTTTCGTGCAGGCAAGGGAGCGGAAATATGAAGGAATGTGTCACAACTGGGAGGGCGCTTCTTCCTGCGGTGAACCCAGGGCAAGAGGCGTGTCTGCACTTCACGGCACCTGGAAGTCCAGACTCAAAATGTCTCAAAATTAAGGTTAAGAGGATCAACCTAAAATGTAAGAAGTCATCATCATATTTTGTTCCTGATGCCCGGTCCAGGTGTACATCAGTGAGGAGATGTCGTTGGGCAGGAGACTGCCAGTCTGGGTGCCCCCCTCATTTCACATCTAACTCCTTTTCTGATGATTGGGCAGGTAAGATGGACAGGGCTGGTCTAGGATTCAGTGGCTGCTCTGATGGATGTGGAGGAGCAGCCTGCGGTTGCTTTAATGCAGCCCCTTCATGCATTTTCTGGAGGAAATGGGTAGAGAATCCACATGGGATCATCTGGAAAGTATCTCCATGTGCTGCATGGGTCCCATCAGCAGTCATAGAGCTAACAATGCCCTCAGGGGAGGTGAGGACATTCCACCCCATGAGCGGAATCCCCACACAAGTCTTCAAGGGTGTGAGTGTAACTTACTTGGGCTCTGATATGGAGGTGTCAGGCTTGACTGACCTGTGTGAGATGGAAGAGCTCAGGTCAAAAAAGCTTGCATTAGCTCCCTGCAACCAGGCTGGTATGGGGGTTGTAGGTAAGGTTGGAGAGATACAGTGCAGTAGCGAGGAAAGTGCCCGTACCATAAAAAAAGATGGGTGCATATGGAATGCTGACCTCGTGGGTATAGAGCTACGAGTGGATGACGCTGTGTGCTACTCTAAGATCACTAGTGTAGAGGCAGTTGCAAACTACTCCGCCATACCCACCACTATTGGGGGGCTGAGGTTTGAGAGAAGCCATGACAGCCAGGGTAAAATATCTGGTAGCCCCCTGGACATCACAGCTATAAGAGGATCTTTTTCTGTCAATTACAGAGGCCTTCGACTGAGTCTCTCAGAAATTACTGCTACTTGTACAGGGGAAGTGACAAATGTGAGTGGGTGTTATTCTTGCATGACAGGCGCCAAAGTCTCCATCAAGTTGCATAGCAGCAAAAATAGCACTGCCCATGTAAGATGCAAAGGGGATGAGACTGCCTTCAGTGTCTTGGAGGGAGTCCATAGCTACTCTGTCAGTCTCAGTTTTGATCATGCAGTAGTTGATGAGCAGTGCCAACTGAACTGTGGAGGACATGAGAGTAAAGTGACTCTAAGAGGCAACCTCATCTTCCTGGATGTCCCGAAATTTGTGGATGGCAGCTACATGCAGACATATCATAGTACAGTGCCCACAGGGGCAAATATCCCAAGCCCTACAGATTGGCTAAATGCCTTGTTTGGCAATGGGCTGAGTAGGTGGTTTCTGGGAGTAATAGGGGTCCTACTGGGGGGATTGGCTCTCTTTTTCTTGATCATGTCTTTGTTCAAGCTGGGAACAAAACAAGTATTTCGATCGAGGTCGAAGCTGGCTTAGATGGGCATATTTCTGGTCTAATGACCCTCTGAGAGCAGTGCTCTCAGGGGAGTTGGCTCACAGTGTGCATGTTTCGTGGTCCTGGCTCACATTCTTAGAGCATGGAGGTTCTATTGAAGTGTTGGCCGGTCTTTGTGT
